# Optimization of Aquaporin Loading for Performance Enhancement of Aquaporin-Based Biomimetic Thin-Film Composite Membranes

**DOI:** 10.3390/membranes12010032

**Published:** 2021-12-27

**Authors:** Yang Zhao, Xuesong Li, Jing Wei, Jaume Torres, Anthony G. Fane, Rong Wang, Chuyang Y. Tang

**Affiliations:** 1 Singapore Membrane Technology Centre, Nanyang Technological University, Singapore 639798, Singapore; zya@aquaporin.com (Y.Z.); xuesong_li@tongji.edu.cn (X.L.); weij0005@e.ntu.edu.sg (J.W.); jtorres@ntu.edu.sg (J.T.); a.fane@unsw.edu.au (A.G.F.); 2 School of Civil and Environmental Engineering, Nanyang Technological University, Singapore 639798, Singapore; 3 School of Biological Sciences, Nanyang Technological University, Singapore 639798, Singapore; 4 Department of Civil Engineering, The University of Hong Kong, Hong Kong 999077, China

**Keywords:** aquaporin, biomimetic thin-film composite membrane, proteoliposome concentration, protein-to-lipid ratio, cholesterol

## Abstract

The aquaporin-based biomimetic thin-film composite membrane (ABM-TFC) has demonstrated superior separation performance and achieved successful commercialization. The larger-scale production of the ABM membrane requires an appropriate balance between the performance and manufacturing cost. This study has systematically investigated the effects of proteoliposome concentration, protein-to-lipid ratio, as well as the additive on the separation performance of ABM for the purpose of finding the optimal preparation conditions for the ABM from the perspective of industrial production. Although increasing the proteoliposome concentration or protein-to-lipid ratio within a certain range could significantly enhance the water permeability of ABMs by increasing the loading of aquaporins in the selective layer, the enhancement effect was marginal or even compromised beyond an optimal point. Alternatively, adding cholesterol in the proteoliposome could further enhance the water flux of the ABM membrane, with minor effects on the salt rejection. The optimized ABM not only achieved a nearly doubled water flux with unchanged salt rejection compared to the control, but also demonstrated satisfactory filtration stability within a wide range of operation temperatures. This study provides a practical strategy for the optimization of ABM-TFC membranes to fit within the scheme of industrial-scale production.

## 1. Introduction

Aquaporins, which serve as water channels in biological membranes [1,2,3], are well known for their ultrahigh permeability to water and near-perfect rejection of any other solute [4,5]. In 2007, Kumar et al. proposed that aquaporin-based biomimetic membranes (ABMs) held great potential to be a next-generation membrane that could offer exceptionally high water permeability and selectivity and was thus capable of breaking the permeability/selectivity trade-off, which has been a persistent challenge for conventional membranes [6]. Since then, a variety of conceptual designs have been proposed and employed to prepare ABMs, such as bio-membrane aperture partition arrays [7,8,9,10,11,12,13], polymer tethered bio-layers [14] and supported bio-membranes with bilayers or vesicles [15,16,17,18,19,20,21,22]. Although some of these designs proved effective to enhance the water flux of resultant ABMs, their long-term performance stability or tolerance to harsh operation conditions were of concern regarding their practical application because of either poor mechanical strength of the resultant membranes or direct exposure of aquaporins to the feed water containing various contaminants. The presence of various contaminants, such as organic matter and heavy metals, may undermine the structure of the protein and cause the deterioration of its function [4]. Furthermore, some of the methods reported for the fabrication of ABMs require a very tedious fabrication process, which is challenging for the large-scale production of ABM membranes. 

To overcome the issues encountered in ABMs’ fabrication and applications, we previously proposed to embed the proteoliposomes (i.e., lipid vesicles reconstituted with aquaporins) entirely into the selective layer of the composite reverse osmosis (RO) or nanofiltration (NF) membrane via interfacial polymerization or crosslinking [23,24]. In comparison with other types of ABMs, the most noticeable characteristic of this configuration is that the proteoliposomes are fully encapsulated in the selective layer, which prevents the fusion or rupture of proteoliposomes and protects the aquaporins from the denaturation effect of contaminants in the feedwater. Long-term filtration tests have demonstrated that the ABM could maintain a relatively stable filtration performance [25]. Compared to other types of ABMs, the ABM based on the configuration of a thin-film composite membrane (ABM-TFC) is much easier to be scaled up, mainly because it negates the need for retrofitting the existing industrial production line of commercial membranes [4,26]. Thanks to these features, the ABM-TFC developed in our lab was quickly commercialized (trademark: Aquaporin Inside) and a series of commercial ABM-TFC membranes such as ABM-RO and ABM-FO have been developed. The commercial ABM-TFC has been widely employed to treat various types of wastewater and demonstrated superior water flux and selectivity with robust mechanical strength [27,28,29,30,31,32,33,34,35,36,37]. Nevertheless, from the perspective of industrial production, the manufacturing cost of membranes is another critical concern, besides the separation performance. Our previous studies demonstrated that the separation performance was strongly correlated with the characteristics of proteoliposomes, or, to be more precise, the loading amount of aquaporin and the characteristics of the proteoliposomes encapsulated in the polyamide layer [23,24,38]. Thus, finding a balance between the manufacturing cost and enhanced separation performance is particularly important for the industrial-scale production of ABM-TFCs, which motivated us to conduct a systematic study on the optimization of aquaporin loading in the fabrication of ABM-TFCs. 

The objective of this work was to systematically study the effects of ABM compositions on ABM separation performance and to identify the optimal preparation conditions for the industrial-scale production of ABM-TFCs. In this study, the effects of different vesicle loadings, protein-to-lipid ratio (PLR) as well as the regulating additive for the proteoliposome (i.e., cholesterol) on the separation performance of ABM-TFCs were systematically investigated. Lastly, the effect of the operation temperature on the water flux and salt rejection of ABM-TFCs (ABM is used to denote the ABM-TFC in short in the following paragraphs of this article) was also examined. 

## 2. Materials and Methods

### 2.1. Chemicals and Materials

#### 2.1.1. Chemicals for Proteoliposome Preparation and Characterization

Analytical-grade NaCl, PBS buffer and sucrose with high purity (>99%) were all purchased from Merck (Singapore). Meanwhile, 1,2-dioleoyl-sn-glycero-3-phosphocholine (DOPC) dissolved in chloroform solution (20 mg lipid/mL) was ordered from Avanti Polar Lipids (Alabaster, AL, USA). n-Octyl-b-D-glucopyranoside (OG, ultrapure grade, Merck, Darmstadt, Germany) was the detergent used for the reconstitution of proteoliposomes. Wild-type AqpZ and AqpZ R189A (inactive mutant) were selected for the preparation of ABMs in this study, and their expression and purification procedure can be found in Section 2.1.3. N-(7-nitrobenz-2-oxa-1,3-diazol-4-yl)-1,2-dihexadecanoyl-sn-glycero-3-phosphoethanolamine, triethylammonium salt (NBD-PE) lipid (fluorescent lipid molecule) and cholesterol were supplied by Invitrogen (Singapore) and Avanti Polar Lipids (Alabama, USA), respectively. Bio-Beads SM2 (Bio-Rad) were used to remove detergent during the reconstitution of proteoliposomes. Ultra-pure water was produced by the Milli-Q^®^ ultrapure water purification system.

#### 2.1.2. Chemicals for Membrane Synthesis and Characterization

Polysulfone (PSf, molecular weight of 75-81 KDa) was supplied by Solvay Advanced Polymers (Winder, GA, USA). N-methyl-2-pyrrolidone (NMP, Merck Pte Ltd, Singapore) was used as the solvent to prepare the polymer dope. Polyvinyl pyrrolidone (PVP, 1300 kDa, Alfa Aesar, Ward Hill, MA, USA) and lithium chloride (LiCl, Sinopharm Chemical Reagent Co. Ltd., Beijing, China) were used as additives for the fabrication of the support. Monomers for interfacial polymerization included trimesoyl chloride (TMC, Sinopharm Chemical Reagent Co. Ltd., Beijing, China) and m-phenylenediamine (MPD, Sigma-Aldrich). Sodium dodecyl sulfate (SDS, Merck Pte Ltd, Singapore) was the additive chosen for the interfacial polymerization. n-hexane (Fisher Scientific Pte Ltd, Singapore) was the organic solvent chosen for the dissolution of TMC. All chemicals were used as received. 

#### 2.1.3. Expression and Purification of AqpZ and Mutant

Both wild-type AqpZ and inactive mutant AqpZ R189A were expressed and used for the proteoliposome reconstitution in this work, while the inactive mutant was used as the control because it preserves a similar structure to the wild-type AqpZ but has significantly lower water permeability [38]. The expression and purification of AqpZ and mutant were done by following the procedure reported in our previous studies [22,23,38]. Briefly, genomic DNA from *E. coli* DH5α was used to amplify the AqpZ or mutant gene, which had gene-specific primers with the addition of a 6-His tag sequence at the N-terminus. The amplified AqpZ or mutant was digested with the enzyme NdeI and BamHI, which was then ligated to the similarly digested pEt3a vector DNA. PCR screening was employed to confirm the positive clones, and the authenticity of the constructs was verified by DNA sequencing (1st base). For the mutant, the arginine residue at position 189 was replaced with alanine to the pET3a/AqpZ by using the Quikchange site-directed mutagenesis kit and the mutation was verified by DNA sequencing (1st base). 

Cell strain C41/pLysS was used for the expression of AqpZ and mutant. Luria broth cultures containing 50 μg/mL ampicillin and 34 μg/mL chloramphenicol were firstly incubated at 37 °C for 16 h, and were then diluted 100-fold and propagated to a proper density (1.2–1.5, OD at 600 nm). Expression of AqpZ or mutant was induced after the addition of 1 mM isopropyl-d-1-thiogalactopyranoside and being kept at 37 °C for 3 h. Harvested cells by centrifugation were resuspended in the binding buffer (20 mM Tris pH 8.0, 50 mM NaCl, 2 mM β-mercaptoethanol, 10 wt% glycerol) at ~4 °C in the presence of 0.4 mg/mL lysozyme, 50 units bensonase and 3 wt% OG. After five lysis cycles in a microfluidizer at 12,000 Pa and centrifugation at 40,000× *g* for 30 min, the supernatant was filtered through a Q-sepharose fast flow column (Amersham Pharmacia GE, Arlington Heights, IL, USA) and the permeate was topped up with 300 mM NaCl before passing through a Ni-NTA column. The Ni-NTA column was washed with a buffer (20 mM Tris pH 8.0, 300 mM NaCl, 50 mM imidazole, 2 mM β-mercaptoethanol, 10 wt% glycerol) to remove non-specifically bound materials. The bound proteins were eluted with the elution buffer (20 mM Tris pH 8.0, 300 mM NaCl, 500 mM imidazole, 2 mM-mercaptoethanol, 10 wt% glycerol, 30 mM OG). The purified wild-type AqpZ and mutant were stocked and kept frozen at 80 °C until further use. The concentration of protein was characterized by UV absorbance at 280 nm with a Nanodrop 1000 spectrophotometer (Thermo Scientific, Ramsey, MN, USA, extinction coefficient = 35,090 M^−1^cm^−1^, molecular weight = 24,524 g/mol).

### 2.2. Reconstitution of Proteoliposomes 

Prior to the reconstitution of proteoliposomes, the liposomes were prepared with the film hydration method. Briefly, an appropriate amount of DOPC lipid or its mixture with cholesterol (with a molar ratio of 30%) or NBD-PE (with a molar ratio of 1%) in chloroform was firstly dried with nitrogen gas, leaving a thin lipid film on the bottom of the vial. The lipid film was thoroughly dried to remove residual chloroform by placing the vial under vacuum for at least 4 h. Next, the lipid film was hydrated with PBS buffer, followed with a vigorous agitation and three cycles of freeze–thawing [39]. Liposomes with uniform size distribution were obtained by extruding the liposome solution through a track-etched polycarbonate filter with a mean pore size of 200 nm 21 times [40]. 

For the reconstitution of proteoliposomes, the protein and liposome suspension were mixed at the desired molar ratio. A certain amount of OG was then added to the mixture and the final concentration of OG was 1 wt%. The detergent was slowly removed by Bio-Beads with 3 changes of 200 mg Bio-Beads per ml of proteoliposomes and incubated at room temperature for 8 h with gentle rotation [22]. Prior to the characterizations or membrane fabrication, the proteoliposome solution was extruded through the track-etched polycarbonate filter with a mean pore size of 200 nm another 15 times. 

### 2.3. Fabrication of ABMs

In this study, a PSf ultrafiltration membrane was prepared for the support of the ABM. The PSf support was fabricated by the phased inversion method described in our previous work [41]. In brief, a mixture containing 15.5 wt% PSf, 81 wt% NMP, 0.5 wt% PVP and 3 wt% LiCl was continuously stirred at 70 °C until a homogenous polymer dope was obtained, which was then degassed in a vacuum desiccator. The nascent PSf membrane was cast on a clean glass plate by using an Elcometer 4340 Motorised Film Applicator (Elcometer Asia Pte Ltd., Singapore) with a gap size of 200 µm, which afterwards was quickly immersed in a coagulant bath (i.e., tap water) to allow the completion of phase inversion. The nascent PSf membrane was kept in tap water for at least 24 h to remove the residual solvent and then stored in Milli-Q water for further use.

The ABM was prepared with a typical interfacial polymerization method [23]. The PSf support was firstly soaked in the proteoliposome-containing MPD solution (1 wt% MPD, 0.1 wt% SDS, an appropriate concentration of proteoliposomes) for 10 min. A compressed nitrogen gas was then applied to remove the excess aqueous solution on the membrane surface. Immediately after removing the aqueous solution, the TMC solution (0.1 wt/vol% in n-hexane) was poured onto the membrane surface to complete the interfacial polymerization reaction. The resultant ABM was stored in Milli-Q water for subsequent characterizations and performance tests. In this article, the ABMs fabricated with different concentrations of proteoliposomes are denoted as ABM-Xw, where X is used to differentiate the concentration of proteoliposomes used in each condition. For example, ABM-8w indicates that the concentration of proteoliposomes in the MPD solution was 8 × 10^−3^ wt% (in terms of the lipid concentration) when preparing this ABM. Likewise, the ABMs fabricated with proteoliposomes having different protein-to-lipid ratios (i.e., the molar ratio of aquaporin molecules to lipid molecules) are denoted as ABM-Yp, where Y represents the protein-to-lipid molar ratio. For instance, ABM-1/400p indicates that the protein-to-lipid molar ratio of proteoliposomes used for preparing this ABM was 1/400. In addition, ABM-0, ABM-M, ABM-C denote the biomimetic membrane containing no proteoliposome, the biomimetic membrane fabricated with the proteoliposome reconstituted with mutants and the biomimetic membrane fabricated with the proteoliposome containing cholesterol, respectively.

### 2.4. Characterizations of Proteoliposomes and ABMs

#### 2.4.1. Size and Water Permeability of Liposomes and Proteoliposomes 

Water permeability of proteoliposomes was measured via stopped-flow experiments, which were performed using a stopped-flow apparatus (SX20, Applied Photophysics, Leatherhead, Surrey, UK). During the stopped-flow experiments, rapid mixing of the proteoliposome solution and a hypo-osmolarity sucrose solution was expected to cause an instant change in the vesicle volume due to water outflux through vesicle bilayers, driven by the osmolarity gradient, which could be monitored by light scattering in real time. The volume change rate, related to the water permeability, could be recorded for permeability calculation [5,6]. The light scattering curve was fitted to a single exponential function with the built-in software (Pro-data SX) to obtain the rate constant *k* [42]. Theoretically, the water permeability of vesicles (*P_f_*) could be calculated by the following equation:

(1) $$P_{f}=\frac{k}{\frac{S}{V_{0}}\cdot V_{w}\cdot \Delta osm}$$

where *S/V_0_* is the ratio between the surface area (*S*) and the initial volume (*V_0_*) of the proteoliposome; *V_w_* is the partial molar volume of water (18 cm^3^/mol), and ∆*osm* is the osmolarity gradient between the intravesicular and extravesicular aqueous solutions. The sizes of proteoliposomes were measured with a Zetasizer (NanoZS, Malvern Instruments Limited, Malvern, UK). Since all proteoliposomes had a similar vesicular size after extrusion through the track-etched membrane (mean pore size: 200 nm), the water permeability of proteoliposomes (*P_f_*) had a nearly linear relationship with the rate constant (Equation (1)). Thus, the rate constant could be used as a crucial parameter to directly correlate the proteoliposome property with the membrane performance [43].

#### 2.4.2. Membrane Characterization

A confocal fluorescence microscope was used to analyze the distribution of proteoliposomes on the PSf support, and the fluorescence images were captured by an LSM 710 system (Carl Zeiss, Oberkochen, Germany) with an excitation wavelength of 488 nm. Before the fluorescence characterization, the PSf support was soaked in MPD solution containing the proteoliposome with 1 mol% fluorescent lipid molecule (i.e., NBD-PE) and the excess aqueous solution on the support surface was removed by compressed nitrogen gas. The cross-sectional scanning electron microscopy (SEM) images of ABMs were acquired by field-emission scanning electron microscopy (FE-SEM, JSM-7600F, JEOL, Akishima, Japan). The membrane samples for SEM analysis were frozen and fractured in liquid nitrogen, and they were then kept in a vacuum desiccator for at least 24 h for the purpose of dehydration. All samples were sputter-coated with gold before SEM characterization. 

#### 2.4.3. Evaluation of the Separation Performance of ABMs

Separation performance of ABMs was investigated in a lab-scale reverse osmosis filtration setup with an operation pressure of 5 bar. A NaCl solution of 10 mM was used as feed and the operation temperature was maintained at 20 ± 0.5 °C. The effective membrane area was 42 cm^2^ and the cross-flow velocity of feed in the membrane cell was ~20 cm/s. Before collecting the permeate for analysis, the membrane samples were pressurized for a sufficiently long time to reach a stabilized state. The water flux (*J*_w,_ L·m^−2^·h^−1^) was determined by measuring the weight of permeate (*m*, kg) during a certain period of time (∆*t*, hour) and could be calculated by the following equation:

(2) $$J_{w}=\frac{m}{\rho \cdot A\cdot \Delta t}$$

where *A* and *ρ* indicate the effective membrane area (m^2^) and the density of water at 20 °C (kg/L), respectively.

Salt rejection (*R*) was calculated by determining the salt concentrations in the feed and permeate based on the conductivities of the feed and the permeate:

(3) $$R=\frac{C_{f}-C_{p}}{C_{f}}$$

where *C_f_* and *C_p_* are the salt concentrations in the feed and the permeate, respectively. The salt permeability coefficient (*B*) can be estimated by the following equation:

(4) $$\frac{1}{R}=\left(1+\frac{B}{J_{w}}\right)$$

## 3. Results and Discussion

### 3.1. Effect of Proteoliposome Concentration on the Characteristics and Separation Performance of ABMs

**Figure 1:**
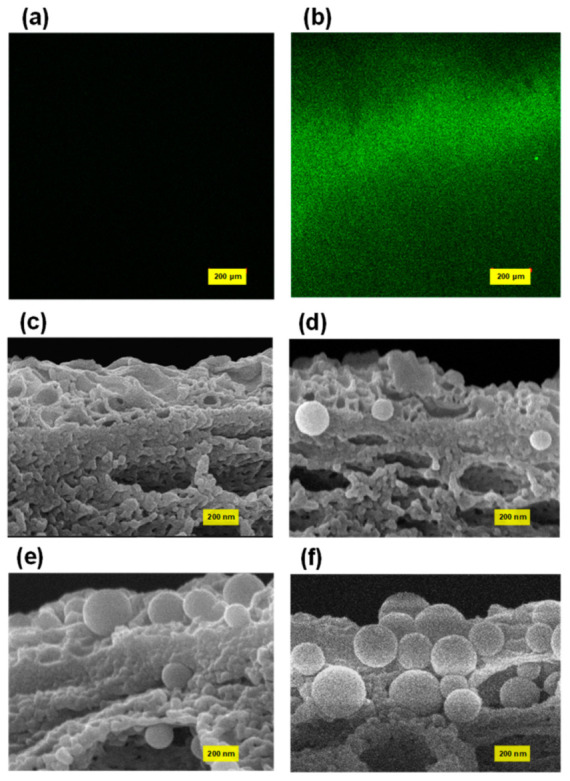
Fluorescence image of (**a**) the PSf support after being soaked with MPD solution and (**b**) the PSf support after being soaked with MPD solution containing fluorescently labelled proteoliposomes. Cross-sectional SEM images of (**c**) ABM-0, (**d**) ABM-8w, (**e**) ABM-16w, (**f**) ABM-32w. All membrane samples for SEM imaging were collected after the filtration performance tests.

In the ABM configuration, the proteoliposome played a pivotal role in increasing the water flux of the ABM membrane as the highly permeable proteoliposomes provided water channels to promote the transport of water molecules through the membrane. As such, the most straightforward protocol to enhance water flux is to increase the loading of proteoliposomes in the polyamide layer. Our previous studies suggested that proteoliposomes exhibited a strong tendency to be attached on the membrane surface even though both proteoliposomes and the PES support carried a negative charge [44,45,46], possibly because of the electrostatic attraction force between the positively charged ionic group of lipids and the negatively charged polymer. We first employed a fluorescence microscope to qualitatively investigate the attachment of proteoliposomes on the PES membrane surface. The control sample, which was immersed in the MPD solution containing no proteoliposome, showed a black background because of the absence of fluorescently dyed proteoliposomes (Figure 1a). In contrast, the fluorescence images (Figure 1b) of the PES membrane that had been immersed in the MPD solution containing fluorescent proteoliposomes showed very strong fluorescent signals over the entire scanned zone, demonstrating a high loading of proteoliposomes on the polymeric support. Consequently, the concentration of proteoliposomes was increased in the aqueous solution for interfacial polymerization for the purpose of increasing the loading amount of proteoliposomes in the polyamide layer. A series of MPD solutions containing different concentrations of proteoliposomes were thus prepared for fabricating ABMs via interfacial polymerization. The FE-SEM was firstly employed to qualitatively characterize the loading amount of proteoliposomes embedded in the polyamide layer by examining the cross-sectional images of the polyamide layers of these ABMs. Through the SEM characterization, the morphology of the polyamide layer as well as the proteoliposomes incorporated in the polyamide layer could be directly visualized. As shown in Figure 1d–f, more proteoliposomes could be discovered in the polyamide layer when more proteoliposomes were loaded in the aqueous solution, suggesting that increasing the concentration of proteoliposomes might be a very effective way to enhance the separation performance of ABMs. Another noticeable characteristic shared by all investigated membrane samples was that the proteoliposomes were fully embedded in the polyamide layer and still remained intact after the polymerization reaction and filtration tests, which could be attributed to the fact that the sufficiently thick polyamide layer (apparent thickness, ~200 nm) encapsulated the proteoliposome and thus enabled it to withstand high hydraulic pressure [23]. 

**Figure 2:**
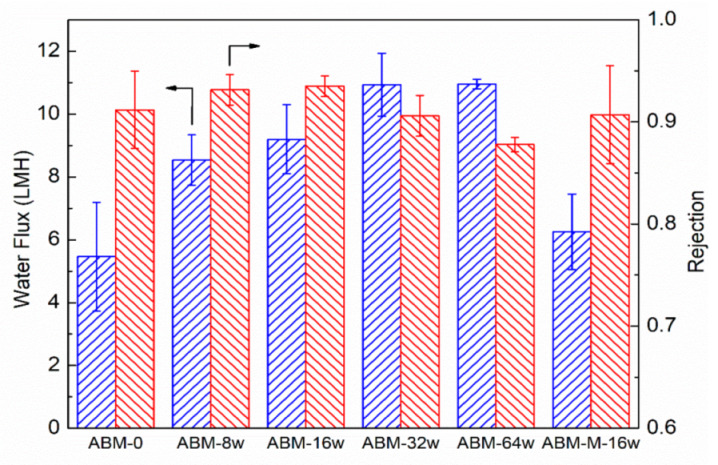
Impacts of the proteoliposome concentration in MPD solution on water flux and salt rejection of the ABM. ABM-M-16w indicates the membrane prepared with the proteoliposome-containing mutant (concentration of proteoliposome was 0.016 wt/wt%). The PLR in all proteoliposomes was 1/200. RO tests were conducted at 5 bar, with 10 mM NaCl solution as a feed solution. The error bars were derived from results for 3 independent samples.

Figure 2 shows the evolution trend of water flux and salt rejection of ABMs with increasing proteoliposome concentration (*l*) in the MPD solution. Even when a low concentration of proteoliposomes (0.008 wt/wt%) was used, the resultant ABM-8w membrane showed a water flux of 8.5 LMH, approximately 60% higher than that (5.4 LMH) of the control (i.e., ABM-0), which was prepared in the absence of proteoliposomes, while salt rejection was also increased from 0.912 to 0.932. When the concentration of proteoliposomes was increased to 0.016 wt/wt%, the water flux and salt rejection of the ABM-16w reached up to 9.2 LMH and 0.935, respectively. Although the salt permeability coefficient of the ABM-16w (0.64 LMH, based on Equation (4)) was slightly higher than that of the control (0.52 LMH), the enhanced salt rejection suggested that the enhancement of water permeation by the proteoliposome was more significant than that of salt permeability (Equation (4)). The increased rejection further validated the formation of an almost defect-free selective layer, as well as the critical role of aquaporin in channeling the transport of water molecules through the PA layer due to its high permeability and selectivity. More water channels reduced the resistance for the transport of water molecules. Meanwhile, the ABM prepared with mutant-incorporated proteoliposomes at the same PLR (200) and concentration (0.016 wt/wt%, ABM-M-16w) showed lower salt rejection (0.907) and water flux (6.3 LMH), which further verified the role of active aquaporin in enhancing water permeation through the polyamide layer. We further increased the concentration of proteoliposomes to 0.032 wt/wt% (ABM-32w) and obtained an even more permeable membrane. However, the increase in water flux was accompanied by a drop in the salt rejection (~0.90). Apparently, the water flux tended to level off when the concentration of proteoliposomes was increased to 0.064 wt/wt% (ABM-64w), but the drop in salt rejection became more noticeable (<0.89). Since an increase in the loading amount of proteoliposomes in the polyamide layer had been confirmed by SEM, it was speculated that agglomeration might occur at a high concentration of proteoliposomes and the oversized agglomerate could disturb the intactness of the polyamide layer during the interfacial polymerization process and cause some defects, resulting in compromised salt rejection. As such, our results demonstrated that introducing AqpZ-containing proteoliposomes with a proper concentration could simultaneously enhance the water flux and salt rejection of ABMs. Therefore, the optimal concentration of proteoliposomes for interfacial polymerization was determined to be 0.016 wt%.

### 3.2. Effect of Protein-to-Lipid Ratio (PLR) on the Separation Performance of ABMs

**Figure 3:**
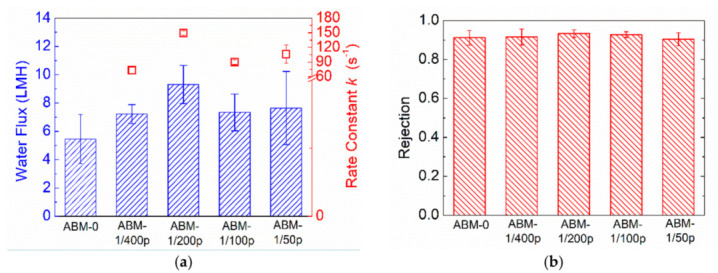
Impacts of PLR on (**a**) water flux and (**b**) rejection of ABM. ABM-0 indicates membranes without incorporation of proteoliposomes. The concentration of proteoliposomes in MPD solution was 0.016 wt% for all AqpZ-containing membranes. RO testing was conducted at 5 bar, with 10 mM NaCl solution as a feed solution. The error bars were derived from results for 3 independent membrane samples.

Other than the proteoliposome concentration, protein-to-lipid ratio (PLR) is another a critical parameter determining the water flux of ABMs. Incorporating more aquaporins in each vesicle is capable of providing more water channels for water permeance. However, a number of studies have shown that an optimal PLR indeed exists when pursuing high water permeability in proteoliposomes. The optimal PLR varies in different studies, possibly because it is highly dependent on the composition of the vesicles, as well as the preparation conditions. The value of rate constant, *k*, reflects the water permeability of proteoliposomes, and the rate constants (Figure 3a) of proteoliposomes with different PLRs measured by the stopped-flow apparatus show that the optimal PLR in our system was 1/200 (~150 s^−1^), which is consistent with the value reported in previous studies [6,38]. Correspondingly, the ABM prepared with the proteoliposomes having a PLR of 1/200 exhibited the highest water permeability (9.3 LMH). The good consistency between the intrinsic water permeability of proteoliposomes and the water permeability of corresponding ABMs further confirms the critical role of proteoliposomes in promoting water transport through the polyamide layer. In terms of rejection, all ABMs exhibited rejection higher than 0.90. The decent selectivity suggested that increasing PLR would not aggravate the agglomeration of proteoliposomes as increasing the proteoliposomes did. 

**Figure 4:**
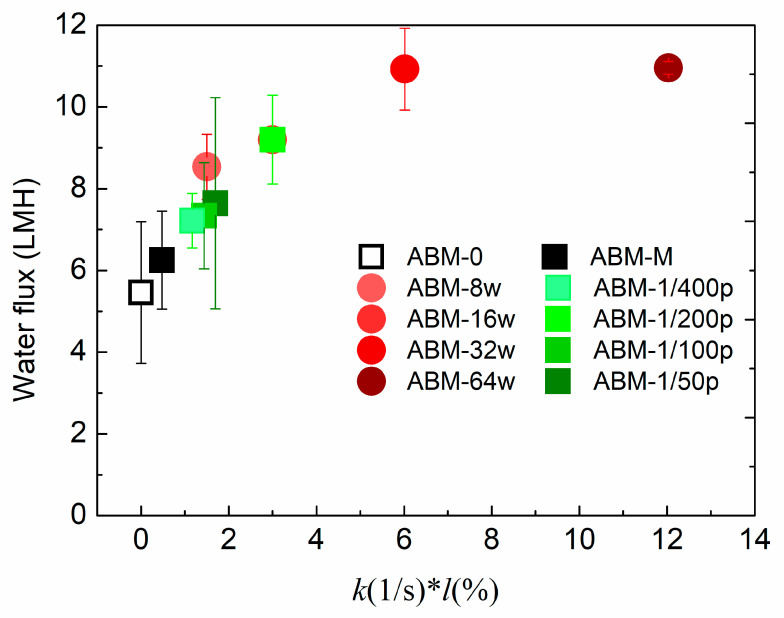
The correlation of water flux of ABMs with the lumped parameter, k*l, where *k, l* indicate rate constant and loading concentration, respectively. Note that ABM-16w is identical to ABM-1/200p since they share the same protein-to-lipid ratio and proteoliposome concentration. *k* values were averaged based on the results of 5 independent measurements.

By investigating the effects of proteoliposome concentration and PLR on the separation performance of ABMs, we could conclude that the proteoliposome concentration and rate constant are the two most important parameters strongly correlated with the separation performance of ABMs. To illustrate the correlation between these two parameters and separation performance, Figure 4 plots the water flux versus a lumped parameter, k*l (unit: *s*^−1^·wt/wt%), where *l* (wt/wt%) represents the proteoliposome concentration in MPD solutions. An obvious trend could be observed whereby increasing k*l resulted in more permeable and selective ABMs when k*l < 4 *s*^−1^·wt/wt%. However, the water flux of the ABM could not be further increased by increasing k*l when k*l >6 *s*^−1^·wt/wt% and, at the same time, salt rejection started to decrease due to the fact that agglomeration might occur at high loading of vesicles. According to the plot shown in Figure 4, it can be inferred that the optimum k*l ranges from 4 *s*^−1^·wt/wt% to 6 *s*^−1^·wt/wt%, which should be targeted when pursuing high water flux and salt rejection. The plot again confirmed that a proper PLR and proteoliposome concentration are pivotal to obtain a highly permeable and selective ABM. In addition, it can be speculated that further enhancing the rate constant at the optimum range might be a more reasonable strategy to increase the water flux of ABMs in comparison with increasing the proteoliposome concentration in the aqueous solution. 

### 3.3. Effect of Cholesterol on ABM Separation Properties

**Figure 5:**
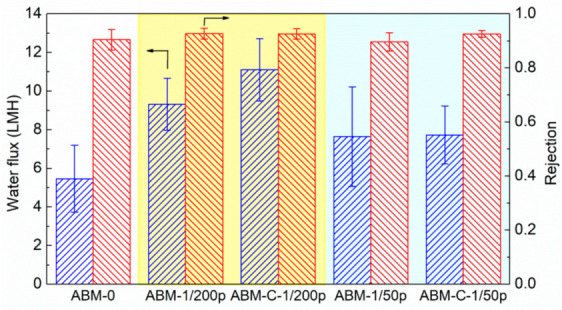
Impacts of the addition of cholesterol in proteoliposomes on the water flux and rejection of the ABM. The molar ratio of cholesterol in the proteoliposome was 30%. The concentration of proteoliposome in the MPD solution was 0.016 wt/wt% for all AqpZ-containing membranes. All tests were performed at 5 bar, with 10 mM NaCl solution as a feed solution. The error bars were derived from results tested with 3 independent membrane samples.

Our previous study showed that introducing cholesterol during the reconstitution process of vesicles could drastically enhance the rate constant of proteoliposomes [43]. Since the water permeability of aquaporin in the vesicle is highly affected by the lipid environment [47], it is speculated that the artificial lipid bilayer containing cholesterol is much closer to the real biomembrane and the addition of cholesterol leads to a more benign lipid environment for AqpZ. The impact of cholesterol on the separation performance of ABMs was also investigated using two types of proteoliposomes with different PLRs (PLR 1/200 and PLR 1/50) in this study. As shown in Figure 5, adding cholesterol significantly enhanced the water flux (from 9.3 LMH to 11.1 LMH) of the ABM prepared with proteoliposomes having a PLR of 1/200, without compromising the salt rejection (0.935 vs. 0.934). However, adding the same amount of cholesterol had little impact on the water flux of the ABM, although it slightly increased the salt rejection, when the PLR of the proteoliposome was 1/50. The different impacts of cholesterol on the water permeability of proteoliposomes and water flux of corresponding ABMs reflected the difference between proteoliposomes in solution and encapsulated proteoliposomes in the PA layer. Nevertheless, adding cholesterol to proteoliposomes can be an effective approach, particularly for industrial-scale production, to further enhance the filtration performance of ABMs given the affordable cost of cholesterol. 

**Figure 6:**
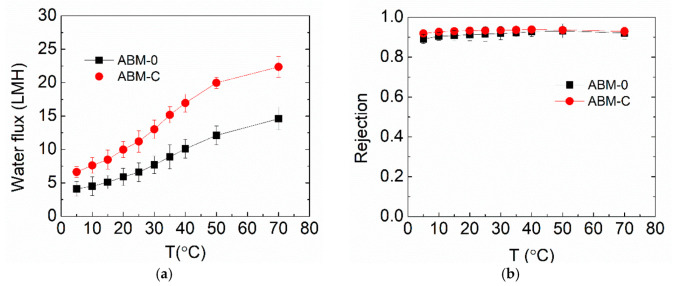
Effects of operation temperature on (**a**) water flux and (**b**) salt rejection of the ABM-C and ABM-0 membranes. The molar ratio of cholesterol in the proteoliposome was 30 % and the concentration of proteoliposomes in the MPD solution was 0.016 wt/wt% for all AqpZ-containing membranes. All tests were performed at 5 bar, with 10 mM NaCl solution as a feed solution. The error bars were derived from results for 2 independent membrane samples.

For real membrane applications, the filtration stability of the optimized ABM is of the utmost importance. In this study, the optimized ABM for RO application was tested at a wide range of temperatures. Figure 6 shows the evolution of the water flux and salt rejection of ABM-0 and ABM-C when increasing the operation temperature from 5 to 70 °C, which certainly covers the operating temperature range of commercial polymeric membranes on the market (generally lower than 45 °C). The water fluxes of both membranes increased noticeably with increasing temperature, while salt rejection only experienced a small increment. This occurred primarily because of the reduced viscosity of water at higher temperatures, allowing water molecules to flow more easily through the membrane. The nearly unchanged salt rejection indicated that the diffusion of salt through the membrane also became faster at higher temperatures, which was probably because the lipid bilayer became more permeable to salts at high temperatures and thus led to higher salt permeability [48]. Nevertheless, the impact of increased salt flux on salt rejection can be offset by the increased water flux, leading to a reduced effect on salt rejection. The optimized ABM demonstrated higher water flux and salt rejection than the control over the entire temperature range, suggesting the good tolerance of proteoliposomes to high temperatures. The good tolerance of proteoliposomes can be attributed to the excellent structural and functional stability of AqpZ at high temperatures [24]. It can be expected that even higher water flux might be reached at elevated temperatures, which could expand the applicability of ABMs in treating those wastewaters with a high temperature, such as radioactive wastewaters. However, this probably requires matrix modifications to ensure a higher temperature tolerance. 

## 4. Conclusions

This study conducted a systematic investigation of the effects of proteoliposome concentration, PLR and cholesterol on the separation performance of ABMs, all of which were found to significantly affect the water flux and salt rejection of ABMs because all related factors, including the loading of proteoliposomes in the polyamide layer, water permeability of proteoliposomes and activity of AqpZ, affected the separation performance of the resultant ABMs. Increasing the proteoliposome concentration could increase the water flux and salt rejection of ABMs within a proper range, while it also deteriorated the membrane performance beyond this. There was an optimal PLR to achieve the highest water flux, and introducing cholesterol at the optimal PLR into proteoliposomes can further enhance the water flux of ABMs. In addition, the optimized ABM showed consistently higher water flux and salt rejection than the control over a wide range of operation temperatures, demonstrating excellent temperature tolerance and robustness. Our study found that an optimal range or condition existed for each of the investigated parameters, which offers a guideline for the determination of a well-balanced point between membrane performance and manufacturing cost for ABMs. Although our study mainly focused on the RO applications, the common characteristics of ABMs make the optimization protocol or conclusions obtained in this study also applicable to other membrane processes such as ABM-FO and ABM-NF.
